# Safety of ACE-I and ARB medications in COVID-19: A retrospective cohort study of inpatients and outpatients in California

**DOI:** 10.1017/cts.2020.489

**Published:** 2020-06-01

**Authors:** Samuel J. S. Rubin, Samuel R. Falkson, Nicholas R. Degner, Catherine A. Blish

**Affiliations:** 1 Department of Medicine, Stanford University School of Medicine, Stanford, CA, USA; 2 Chan-Zuckerberg Biohub, San Francisco, CA, USA

**Keywords:** COVID-19, SARS-CoV-2, ACE inhibitors (ACE-I), translational science, angiotensin II receptor blockers (ARB), hypertension, renin-angiotensin-aldosterone system (RAAS)

## Abstract

**Introduction:**

There is significant interest in the use of angiotensin converting enzyme inhibitors (ACE-I) and angiotensin II receptor blockers (ARB) in coronavirus disease 2019 (COVID-19) and concern over potential adverse effects since these medications upregulate the severe acute respiratory syndrome coronavirus 2 host cell entry receptor ACE2. Recent studies on ACE-I and ARB in COVID-19 were limited by excluding outpatients, excluding patients by age, analyzing ACE-I and ARB together, imputing missing data, and/or diagnosing COVID-19 by chest computed tomography without definitive reverse transcription polymerase chain reaction (RT-PCR), all of which are addressed here.

**Methods:**

We performed a retrospective cohort study of 1023 COVID-19 patients diagnosed by RT-PCR at Stanford Hospital through April 8, 2020 with a minimum follow-up time of 14 days to investigate the association between ACE-I or ARB use with outcomes.

**Results:**

Use of ACE-I or ARB medications was not associated with increased risk of hospitalization, intensive care unit admission, or death. Compared to patients with charted past medical history, there was a lower risk of hospitalization for patients on ACE-I (odds ratio (OR) 0.43; 95% confidence interval (CI) 0.19–0.97; *P* = 0.0426) and ARB (OR 0.39; 95% CI 0.17–0.90; *P* = 0.0270). Compared to patients with hypertension not on ACE-I or ARB, patients on ARB medications had a lower risk of hospitalization (OR 0.09; 95% CI 0.01–0.88; *P* = 0.0381).

**Conclusions:**

These findings suggest that the use of ACE-I and ARB is not associated with adverse outcomes and may be associated with improved outcomes in COVID-19, which is immediately relevant to care of the many patients on these medications.

## Introduction

Severe acute respiratory syndrome coronavirus 2 (SARS-CoV-2) has spread across the globe, causing a WHO designated pandemic of coronavirus disease 2019 (COVID-19). While vaccines and antiviral medications are being developed, significant interest has also centered around expedited repurposing of approved medications. Controversy has surrounded the use of renin–angiotensin–aldosterone system (RAAS) inhibitors, including angiotensin converting enzyme inhibitors (ACE-I) and angiotensin II receptor blockers (ARB), in the setting of COVID-19. While numerous papers have proposed potential benefits and risks of ACE-I and ARB in the management of COVID-19,^1–7^ data are still lacking in two key areas: (i) whether outpatient use of these drugs is associated with risk of hospitalization and (ii) differentiating their individual class effects (ACE-I versus ARB) on outcomes in light of their distinct mechanisms of action.^8–10^ Furthermore, several of these recent ACE-I/ARB studies identified some COVID-19 patients by chest computed tomography (CT) without definitive reverse transcription polymerase chain reaction (RT-PCR) testing, excluded some patients based on age, and/or imputed missing data. Clinical trials are underway to assess the efficacy of ACE-I and ARB medications in treatment of COVID-19 (e.g., NCT 04330300, 04312009, 04311177, and others), but these will require significant time to yield conclusions. In the meantime, direct record-based data on separate ACE-I and ARB medications in inpatient and outpatient populations are needed to evaluate associations between their use and COVID-19 severity.

SARS-CoV-2 binds ACE2 to enter host cells, leading to significant interest in the role of the RAAS pathway in COVID-19 disease.^11,12^ ACE2 promotes an anti-inflammatory state, which could be beneficial in the setting of COVID-19. Use of ACE-I and ARB is associated with upregulation of ACE2 in animals and some human studies^13^ and has also demonstrated benefit in animal models and small human studies of sepsis and lung injury due to viral infections.^14–20^ Several experts have suggested that ACE-I and/or ARB use could limit COVID-19-associated inflammatory damage, while others have cautioned that resultant upregulation of ACE2 could enhance host cell viral entry and even suggested discontinuation of these medications.^8,21^ On the other hand, ACE-I- and ARB-mediated upregulation of ACE2 could augment not only membrane-bound but also soluble ACE2, which may act as a decoy receptor to saturate SARS-CoV-2 virions and modulate host cell viral entry, representing a potential anti-viral mechanism.^4,22^ Moreover, there could be risk of discontinuing ACE-I and ARB, particularly in the outpatient setting, given the benefits of these therapies in treating conditions such as hypertension and heart failure.^23^ Finally, while studies to date have grouped COVID-19 patients on ACE-I or ARB medications together, it is likely that these therapies could have distinct effects in the setting of COVID-19 given their different mechanisms of action, and therefore, they should be analyzed independently. As ACE-I and ARB medications are used widely among COVID-19 susceptible individuals, it is important to rigorously evaluate the impact of ACE-I and ARB use on rates of hospitalization and disease outcomes in COVID-19.^24,25^

A significant portion of individuals with COVID-19 has risk factors consistent with eligibility for ACE-I and/or ARB use.^26,27^ To assess the effects of ACE-I and ARB in COVID-19 patients on incidence of hospitalization and disease course, we studied the effects of ACE-I and ARB separately in a diverse cohort of COVID-19 inpatients and outpatients regarding the safety and potential benefit of ACE-I or ARB use in the setting of COVID-19 using electronic medical record data.

## Methods

### Study Population

With approval of the Stanford Institutional Review Board, patient charts were analyzed if they were diagnosed with COVID-19 by RT-PCR and received care at Stanford Hospital and Clinics through April 8, 2020. A total of 1023 patients met these criteria, including inpatients and outpatients.

### Statistics

Statistical analyses were conducted using Microsoft Excel, R, GraphPad Prism 8, and ClinCalc.com, *P* < 0.05 was considered statistically significant, and all statistical tests were two-sided. Odds ratios (ORs) were calculated using the Baptista–Pike method. E-values were calculated using the formula, 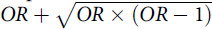, where the inverse odds ratio was used when OR < 1. To estimate the number of patients required per group to detect an effect of ACE-I or ARB use on hospitalization among all patients with past medical history, a power calculation was performed using alpha = 0.05, beta = 0.20, a 33.3% hospitalization rate based on a previously published cohort from Stanford Hospital,^7^ a 60% decrease in incidence, which indicated a minimum of 32 individuals per ACE-I and 33 individuals per ARB group with enrollment ratios of 11.3:1 and 9.0:1 for non-ACE-I and non-ARB users, respectively. All RT-PCR test-positive COVID-19 patients who received care at Stanford Hospital were included in the study through April 8, 2020, after which there were 48 patients on ACE-I medications and 49 patients on ARB medications. Subsequently, all patients were followed through resolution of COVID-19, or at minimum of 14 days after presentation, as suggested by the upper bound of the interquartile range for length of hospitalization in a study by Guan *et al.*, 2020 and by the expected time required for progression to COVID-19 pneumonia.^26,27^

### Data Definitions

Routinely collected clinical data were recorded in a standardized manner before group stratification or analysis to minimize the effects of bias, and all collected data were based on explicit documentation in the chart as determined by manual review. Missing data were not imputed. Patients without documentation of certain features were excluded from analysis of those specific features to avoid data skewing. Race/ethnicity was categorized as African American, Asian, Hispanic, Pacific Islander, White, or unknown. Pre-existing diagnoses selected for collection were based on documentation of past medical history. Type I diabetes and type II diabetes were all included in defining history of diabetes, but prediabetes and gestational diabetes were excluded. Last available body mass index (BMI) values were recorded. Admission to the hospital was defined by all cause admission. The primary cause of admission for 123 patients was COVID-19; 12 additional patients were admitted for other primary causes, which may have been related to COVID-19, two for altered mental status (AMS), one for AMS secondary to metabolic or septic encephalopathy, one for hypercapnea resulting in AMS, one for anti-N-methyl-D-aspartate receptor autoimmune encephalitis, one for hyponatremia, one for pancytopenia, one for C-section delivery, one for hip fracture, one for urinary tract infection, one for acute cholecystitis, and one for fever. Sequential Organ Failure Assessment (SOFA) and Acute Physiology And Chronic Health Evaluation II (APACHE II) illness scores were calculated using mdcalc.com for patients admitted to the intensive care unit (ICU) with available requisite data based on the most extreme measurements in the first 24 hours after ICU admission or first available measurements if there were multiple measurements and they were within the normal standard reference range; a perfect Glasgow Coma Score of 15 was recorded for patients with a normal neurological exam. As a surrogate marker of disease severity, maximum oxygen requirements were recorded in ascending order: room air, nasal cannula (NC), high-flow nasal cannula (HFNC), bilevel or continuous positive airway pressure (CPAP), and intubation. History of CPAP use for obstructive sleep apnea was not included in the positive airway pressure (PAP) category. Laboratory values were recorded as first available at presentation. History of smoking was only determined based on explicit documentation; if smoking status was not documented in the chart, then these patients were excluded from analysis of smoking.

## Results

### Study Population

**Fig. 1.**
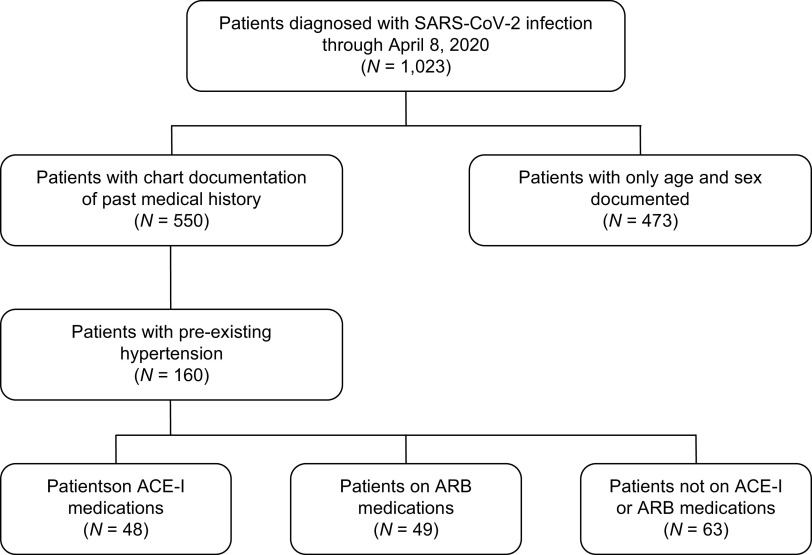
Flowchart of patient identification and stratification. A total of 1,023 patients were identified as being infected with SARS-CoV-2 by RT-PCR at Stanford Hospital and were diagnosed with COVID-19. Of these COVID-19 patients, 550 had chart documentation of past medical history, and 160 had pre-existing diagnoses of hypertension. Of patients with hypertension, 48 were on ACE-I medications and 49 were on ARB medications. ACE-I, angiotensin converting enzyme inhibitors; ARB, angiotensin II receptor blockers.

Patients who were diagnosed with COVID-19 by RT-PCR and received care at Stanford Hospital and Clinics through April 8, 2020 were included in the study and followed through April 22, 2020, at minimum 14 days from presentation (Fig. 1; Table 1). All analysis was based on chart documentation, and no missing data were imputed. The cohort was diverse, with the youngest patient being 6 months old and the oldest 100 years old. Consistent with previous studies,^25^ the most common pre-existing diagnosis among patients over 18 years of age was hypertension (29.1%), followed by diabetes (16.0%). Of patients with hypertension, 48 (30.0%) were on ACE-I medications, and 49 (30.6%) were on ARB medications.

**Table 1.** Baseline characteristics and clinical outcomes of patients in the study

Baseline characteristics	Median (interquartile range) or *N* (%)^a^									
			No ACE-I or ARB			*p*-Values^b^				
	ACE-I (*N* = 48)	ARB (*N* = 49)	Patients with HTN (*N* = 63)	Patients with PMH (*N* = 453)	Patients without PMH (*N* = 473)	ACE-I vs. ARB	ACE-I vs. HTN	ARB vs. HTN	ACE-I vs. PMH	ARB vs. PMH
Age	63.0 (19.5, 33.0–96.0)	70.0 (19.0, 39.0–95.0)	65.0 (25.0, 29.0–100.0)	45.0 (26.0, 0.8–100)	52.0 (32.0, 0.5–96.0)	0.0605	0.4102	0.4054	5.4076E–11	1.0000E–15
Female sex	27 (56.3%)	22 (44.9%)	31 (49.2%)	235 (51.9%)	201 (42.5)	0.3123	0.5655	0.7051	0.6491	0.3705
Race/ethnicity										
African American	4 (8.3%)	2 (4.1%)	2 (3.2%)	11 (2.4%)		0.4357	0.3999	1.0000	0.0460	0.3678
Asian	4 (8.3%)	13 (26.5%)	4 (6.3%)	62 (13.7%)		0.0307	0.7247	0.0065	0.3742	0.0320
Hispanic	15 (31.3%)	13 (26.5%)	19 (30.2%)	101 (22.3%)		0.6585	1.0000	0.8333	0.2062	0.4778
Pacific Islander	1 (2.1%)	1 (2.0%)	1 (1.6%)	7 (1.5%)		1.0000	1.0000	1.0000	0.5559	0.5630
White	13 (27.1%)	12 (24.5%)	26 (41.3%)	169 (37.3%)		0.8194	0.1604	0.0727	0.2065	0.0856
Body mass index	31.0 (9.6, 16.7–44.0)	28.2 (5.1, 21.2–47.9)	28.0 (8.8, 19.7–54.8)	26.2 (7.7, 15.4–54.9)		0.3161	0.4292	0.9657	0.0052	0.0175
Pre-existing diagnoses										
Diabetes	23 (47.9%)	28 (57.1%)	19 (30.2%)	37 (8.2%)		0.4188	0.0754	0.0066	3.4819E–11	2.0000E–15
Asthma	4 (8.3%)	7 (14.3%)	4 (6.3%)	51 (11.3%)		0.5240	0.7247	0.2065	0.8072	0.4852
Cancer	9 (18.8%)	7 (14.3%)	17 (27.0%)	44 (9.7%)		0.5947	0.3697	0.1629	0.0785	0.3186
Coronary artery disease	5 (10.4%)	14 (28.6%)	14 (22.2%)	17 (3.8%)		0.0391	0.1298	0.5118	0.0493	8.8042E–08
Autoimmune or autoinflammatory	3 (6.3%)	3 (6.1%)	5 (7.9%)	21 (4.6%)		1.0000	1.0000	1.0000	0.4930	0.7200
Heart failure	4 (8.3%)	6 (12.2%)	11 (17.5%)	15 (3.3%)		0.7404	0.2621	0.5971	0.0981	0.0111
Chronic obstructive pulmonary disease	1 (2.1%)	3 (6.1%)	4 (6.3%)	8 (1.8%)		0.6171	0.3869	1.0000	0.5991	0.0824
History of any smoking	16 (33.3%)	18 (36.7%)	24 (38.1%)	83 (18.3%)		0.8321	0.6913	1.0000	0.0207	0.0043
Selected labs at presentation										
Potassium (mmol/l)	4.2 (0.7, 3.2–5.4)	4.0 (0.6, 2.9–4.6)	3.9 (0.7, 3.3–5.20	3.9 (0.8, 2.5–6.1)		0.3829	0.4386	0.9727	0.1204	0.5369
Sodium (mmol/l)	137.0 (3.8, 129.0–144.0)	135.0 (9.5, 111.0–144.0)	138 (5.0, 124.0–146.0)	137.0 (5.0, 118.0–146.0)		0.0436	0.8742	0.0324	0.2813	0.1000
Creatinine (mg/dl)	1.0 (0.5, 0.4–2.5)	0.9 (0.2, 0.5–8.6)	1.1 (0.9, 0.6–7.3)	0.8 (0.3, 0.5–7.3)		0.5337	0.1202	0.0184	0.2712	0.6738
Outcome										
Time from onset to presentation (days)	4.5 (4.0, 1.0–17.0)	5.0 (4.0, 0.0–29.0)	4.0 (5.0, 0.0–22.0)	5.0 (5.0, –8.0–31.0)						
Admitted to hospital	15 (31.3%)	21 (42.9%)	31 (49.2%)	99 (21.9%)						
Admitted to ICU	5 (10.4%)	11 (22.9%)	9 (14.8%)	31 (6.9%)						
SOFA score^c^	6.0 (0.0, 6.0–6.0)	2.0 (0.0, 2.0–2.0)	2.5 (0.5, 2.0–3.0)	3.7 (2.5, 2.0–6.0)						
APACHE II score^c^	18.0 (0.0, 18.0–18.0)	15.0 (5.5, 5.0–16.0)	5.0 (0.0, 5.0–5.0)	10.0 (5.0, 5.0–20.0)						
Maximum oxygen requirement										
Room air	5 (41.7%)	5 (25.0%)	9 (34.6%)	38 (42.2%)						
Nasal cannula	5 (41.7%)	7 (35.0%)	8 (30.8%)	27 (30.0%)						
High-flow nasal cannula	0 (0%)	3 (15.0%)	4 (15.4%)	8 (8.9%)						
Positive airway pressure	0 (0%)	0 (0%)	0 (0%)	1 (1.1%)						
Intubation	2 (16.7%)	5 (25.0%)	5 (19.2%)	16 (17.8%)						
Length of intubation (days)	10.0 (7.0, 3.0–17.0)	9.0 (7.0, 2.0–16.0)	10.0 (0.0, 10.0–10.0)	13.5 (5.5, 10.0–17.0)						
Length of hospital stay (days)	5.5 (8.8, 1.0–29.0)	6.5 (9.3, 1.0–21.0)	10.0 (6.0, 2.0–34.0)	6.0 (8.0, 1.0–34.0)						
Remaining in hospital	0 (0%)	2 (4.1%)	4 (6.3%)	12 (2.6%)						
Death	4 (8.3%)	2 (4.1%)	6 (9.5%)	12 (2.6%)						

ACE-I, angiotensin converting enzyme inhibitor; APACHE II, Acute Physiology And Chronic Health Evaluation II; ARB, angiotensin II receptor blockers; HTN, hypertension; ICU, intensive care unit; PMH, past medical history; SOFA, Sequential Organ Failure Assessment.

a Percentages were calculated using number of patients in the group (column) with available data for the corresponding parameter (row).

b *p*-Values from Fisher’s exact test for categorical variables or Mann–Whitney U test for continuous variables.

c Scores calculated for patients admitted to the ICU when data available (SOFA: *n* = 2 patients on ACE-I, 2 on ARB, 2 with HTN, and 10 with PMH; APACHE II: *n* = 1 on ACE-I, 3 on ARB, 1 with HTN, and 11 with PMH).

To confirm that selection of patients on ACE-I and ARB was not confounded by additional risk factors, we compared baseline characteristics of patients on either medication to the other patients with hypertension or the other patients with past medical history (Table 1). The median age was 63 for patients on ACE-I and 70 for patients on ARB, but not significantly different. There was a significantly higher representation of Asian race/ethnicity, history of coronary artery disease (CAD), and lower serum sodium concentration at presentation among patients on ARB medications compared to ACE-I medications. There were no significant differences between patients on ACE-I medications and other patients with hypertension. Patients on ACE-I medications were significantly older, had a higher representation of African American race/ethnicity, higher BMI, more history of diabetes, more history of cancer, more history of CAD, more history of heart failure, more smoking history, and higher serum creatinine concentrations at presentation compared to other patients with past medical history. There was a significantly higher representation of Asian race/ethnicity, more history of diabetes, and lower serum sodium and creatinine concentrations at presentation among patients on ARB medications compared to other patients with hypertension. Patients on ARB medications were significantly older, had higher BMI, more history of diabetes, more history of CAD, more history of heart failure, more history of chronic obstructive pulmonary disease, and more history of smoking compared to other patients with past medical history. These findings informed subsequent multivariable modeling of baseline characteristics with outcomes to account for potentially confounding variables. Since patients on ACE-I or ARB medications were more likely to have risk factors associated with worse outcomes than the comparator groups based on prevous studies, it is unlikely that selection of patients on ACE-I or ARB medications by baseline characteristics would enhance associations between use of ACE-I or ARB and reduced risk of the outcomes measured.

### Outcomes

The median time from onset of symptoms to presentation was 5 days (Table 1). Of 550 patients with documented past medical history, 135 (24.5%) were admitted to the hospital based on data available in the Care Everywhere software network, 47 (8.5%) were admitted to the ICU, and 18 (3.3%) died. The median length of hospital stay was 6 days, and at the time, this study was completed 14 patients remained in the hospital. Disease severity was determined by SOFA and APACHE II illness scores as well as maximum oxygen requirement, ranging in ascending order of severity from room air, to NC, HFNC, PAP, or intubation. Of hospitalized patients, 48 (39.3%) required room air, 39 (32.0%) NC, 11 (9.0%) HFNC, 1 (0.8%) PAP, and 23 (18.9%) intubation.

### Association of Baseline Characteristics and ACE-I or ARB Medication Use With Outcomes

We analyzed baseline characteristics among patients with documentation of past medical history to identify risk factors for outcomes of admission to the hospital, admission to the ICU, and death (Table 2). A stepwise approach was used to identify risk factors independently associated with outcomes and to avoid overfitting the multivariable model. This workflow included univariate significance tests of each potential risk factor (baseline characteristic) with each outcome (level 1), followed by multivariable logistic regression of each factor significant at level 1 as well as ACE-I and ARB medications controlling for age (an expected strong confounder) with each corresponding outcome (level 2, not shown), and finally multivariable logistic regression of all factors significant at level 2 as well as ACE-I (a) or ARB (b) with age and each corresponding outcome (levels 3a and 3b). When patients on ACE-I medications were compared to those not on ACE-I medications, patients on ARB medications were excluded from the analysis, and vice versa.

**Table 2.** Association of baseline characteristics with clinical outcomes amongst 550 patients with documented past medical history.

	Univariate^a^						Multivariable^b^											
	Admission to hospital		Admission to ICU		Death		ACE-I + confounders						ARB + confounders					
Baseline characteristic	p-value	OR or difference of medians (95% CI)^c^	p-value	OR or difference of medians (95% CI)^c^	p-value	OR or difference of medians (95% CI)^c^	Admission to hospital	OR (95% CI)^c^	Admission to ICU	OR (95% CI)^c^	Death	OR (95% CI)^c^	Admission to hospital	OR (95% CI)^c^	Admission to ICU	OR (95% CI)^c^	Death	OR (95% CI)^c^
Age	1.0000E-15	17.00 (12.00-20.00)	6.2689E-05	12.00 (6.00-18.00)	4.8276E-08	29.00 (18.00-35.00)	4.2800E-07	1.04 (1.02-1.06)	0.6013	1.01 (0.98-1.03)	0.0030	1.06 (1.02-1.10)	4.8400E-08	1.04 (1.03-1.06)	0.3287	1.01 (0.99-1.04)	0.0039	1.07 (1.02-1.11)
Female sex	0.1973	0.76 (0.52-1.13)	0.2218	0.66 (0.39-1.22)	0.0530	0.35 (0.14-0.93)												
Body mass index	0.0062	1.75 (0.50-2.70)	0.0001	3.60 (1.60-4.90)	0.6484	1.10 (-2.20-3.40)	0.1030	1.03 (0.99-1.07)	0.0059	1.08 (1.02-1.14)			0.0041	1.06 (1.02-1.10)	0.0057	1.08 (1.02-1.14)		
Pre-existing diagnoses																		
Hypertension	7.7132E-09	3.40 (2.25-5.09)	3.1354E-05	3.54 (1.96-6.29)	0.0009	5.18 (1.99-13.67)												
Diabetes	6.6835E-10	4.58 (2.84-7.43)	4.2427E-09	7.09 (3.78-13.36)	0.0007	5.72 (2.35-13.79)	9.5800E-05	3.99 (1.99-7.99)	9.8900E-06	7.33 (3.03-17.73)			0.0002	3.84 (1.90-7.76)	0.0002	4.88 (2.11-11.28)		
Asthma	0.5346	0.79 (0.41-1.47)	0.3405	0.51 (0.16-1.53)	0.4436	0.63 (0.18-2.09)												
Cancer	0.0022	2.46 (1.42-4.27)	0.0007	3.68 (1.82-7.52)	0.0002	7.37 (2.79-20.24)			0.0351	2.89 (1.08-7.77)					0.0416	2.60 (1.04-6.54)		
Coronary artery disease	2.4592E-07	6.33 (3.18-12.73)	0.0003	5.12 (2.34-11.11)	2.5822E-07	18.67 (7.11-48.37)	0.2605	1.85 (0.63-5.38)	0.9517	1.04 (0.27-4.01)	0.0023	7.77 (2.08-28.95)	0.0192	3.13 (1.20-8.11)	0.2489	1.89 (0.64-5.59)	0.0036	8.50 (2.01-35.87)
Autoimmune or autoinflammatory	0.0096	3.04 (1.43-6.34)	0.4985	1.35 (0.41-4.15)	0.0528	4.23 (1.22-13.84)	0.0979	2.25 (0.86-5.89)					0.0284	2.91 (1.12-7.58)				
Heart failure	0.0001	5.05 (2.32-11.21)	0.4664	1.48 (0.45-4.62)	0.0067	6.94 (2.31-20.56)												
Chronic obstructive pulmonary disease	0.0022	6.46 (1.83-19.49)	0.0143	5.71 (1.84-17.77)	0.0552	6.53 (1.74-32.53)												
History of any smoking	0.0052	1.93 (1.22-2.98)	0.2800	1.44 (0.75-2.72)	0.152	2.15 (0.83-5.58)												
Medications																		
ACE-I^d^	0.1490	1.63 (0.87-3.15)	0.3747	1.58 (0.64-4.24)	0.0571	3.34 (1.14-11.02)	0.0426	0.43 (0.19-0.97)	0.1109	0.38 (0.12-1.25)	0.4840	1.60 (0.43-5.93)						
ARB^d^	0.0023	2.68 (1.45-4.97)	0.0009	4.03 (1.79-8.66)	0.6372	1.56 (0.34-6.51)							0.0270	0.39 (0.17-0.90)	0.8948	1.07 (0.41-2.81)	0.2063	0.34 (0.06-1.82)

a p-values from Fisher’s exact test for categorical variables or Mann-Whitney U test for continuous variables.

b p-values from logistic regression.

c OR indicates ratio of outcome in presence compared to absence of categorical baseline characteristic; difference of medians indicates median of outcome-positive group minus median of outcome-negative group for continous variables.

d patients on ARB were excluded from the group compared to patients on ACE-I, and patients on ACE-I were excluded from the group compared to patients on ARB; OR, odds ratio; CI, confidence interval.

Among all patients with chart documentation of past medical history, age was independently significantly associated with admission to the hospital and with death, but not with admission to the ICU (Table 2). Diabetes was independently significantly associated with admission to the hospital and to the ICU. BMI and history of cancer were independently significantly associated with admission to the ICU. CAD was independently significantly associated with death. Use of ACE-I medications was associated with a reduced risk of hospital admission (OR 0.43; 95% confidence interval (CI) 0.19–0.97; *P* = 0.0426), but not ICU admission or death. Use of ARB medications was associated with a reduced risk of hospital admission (OR 0.39; 95% CI 0.17–0.90; *P* = 0.0270), but not ICU admission or death. Calculation of E-values to indicate potential unmeasured confounding supports the strength of these results (Supplementary Table 1). These data suggest that ACE-I and ARB medications are associated with a reduced risk of hospitalization and no increased risk of admission to the ICU or death in the setting of COVID-19.

### Association of ACE-I or ARB Medication Use With Outcomes Among Patients With Hypertension

Focusing on the 160 patients with the most common pre-existing diagnosis of hypertension, we analyzed the association of baseline and presenting risk factors with outcomes of admission to the hospital, admission to the ICU, and death, as well as maximum oxygen requirements using the aforementioned stepwise approach (Table 3). No hypertension patients required PAP as their maximum oxygen requirement, and five remained hospitalized at the time this study was completed. Of the 48 patients on ACE-I medications, 30 were also on another hypertension medication. Of the 49 patients on ARB medications, 31 were also on another hypertension medication. In the context of their COVID-19 disease management, ACE-I medication was withheld in eight patients and ARB medication was withheld in seven patients. When patients on ACE-I medications were compared to those not on ACE-I medications, patients on ARB medications were excluded from the analysis, and vice versa.

**Table 3.** Association of baseline characteristics with clinical outcomes amongst 160 patients with hypertension.

	Univariate^a^												Multivariable^b^											
	Admission to hospital		Admission to ICU		Death		Maximum oxygen requirement^d^						ACE-I + confounders						ARB + confounders					
							Nasal cannula		HFNC		Intubation		Admission to hospital	OR or difference of medians (95% CI)^c^	Admission to ICU	OR or difference of medians (95% CI)^c^	Death	OR or difference of medians (95% CI)^c^	Admission to hospital	OR or difference of medians (95% CI)^c^	Admission to ICU	OR or difference of medians (95% CI)^c^	Death	OR or difference of medians (95% CI)^c^			
Baseline characteristic	p-value	OR or difference of medians (95% CI)^c^	p-value	OR or difference of medians (95% CI)^c^	p-value	OR or difference of medians (95% CI)^c^	p-value	OR or difference of medians (95% CI)^c^	p-value	OR or difference of medians (95% CI)^c^	p-value	OR or difference of medians (95% CI)^c^												
Age	2.6739E-05	13.00 (5.00-15.00)	0.8326	0.00 (-6.00-8.00)	0.0009	16.00 (6.00-22.00)	0.2921	-4.00 (-16.00-6.00)	0.6818	4.00 (-19.00-10.00)	0.1607	-5.50 (-15.00-4.00)	0.0117	1.09 (1.02-1.17)	0.1243	0.97 (0.93-1.01)	0.2468	1.04 (0.94-1.11)	0.0038	1.11 (1.04-1.20)	0.5359	0.99 (0.95-1.03)	0.2071	1.05 (0.97-1.13)
Female sex	0.7487	1.17 (0.63-2.18)	0.5204	0.74 (0.32-1.76)	0.1307	3.25 (0.83-11.45)	0.5006	0.57 (0.15-2.50)	0.1904	4.29 (0.72-24.55)	0.1489	3.43 (0.81-12.77)												
Body mass index	0.4189	-1.00 (-1.20-2.50)	0.1208	3.30 (-0.50-4.40)	0.2068	2.05 (-1.60-5.70)	0.0915	2.45 (-0.40-7.20)	0.2495	2.60 (-2.10-7.90)	0.2997	1.05 (-2.00-5.80)												
Pre-existing diagnoses																								
Diabetes	0.0061	2.50 (1.32-4.62)	0.0038	3.96 (1.57-9.95)	0.1313	2.77 (0.88-8.54)	0.7524	1.36 (0.40-4.91)	0.1783	6.67 (0.66-82.97)	0.7160	1.56 (0.40-7.16)	0.0311	8.91 (1.22-65.05)	0.0042	7.52 (1.89-29.87)			0.1509	4.48 (0.58-34.70)	0.0306	3.46 (1.12-10.65)		
Asthma	0.7857	1.24 (0.47-3.71)	1.0000	0.80 (0.17-3.56)	0.0919	3.69 (0.96-14.61)	0.6614	0.59 (0.10-3.25)	0.5396	0.00 (0.00-3.13)	1.0000	0.48 (0.04-3.71)												
Cancer	0.4314	1.40 (0.63-3.08)	0.1793	2.01 (0.78-4.97)	0.0732	3.06 (1.00-9.76)	0.4075	0.42 (0.07-2.09)	1.0000	1.50 (0.23-10.38)	0.6757	1.88 (0.44-7.89)												
Coronary artery disease	0.0056	3.08 (1.40-7.03)	0.0546	2.67 (1.11-6.35)	0.0004	9.84 (3.03-30.57)	0.7164	1.61 (0.41-5.83)	0.3401	2.81 (0.51-19.19)	0.1271	3.75 (0.79-15.26)					0.0061	11.46 (2.00-65.53)			0.2114	2.04 (0.67-6.24)	0.0154	17.50 (1.73-117.25)
Autoimmune or autoinflammatory	0.2039	2.60 (0.77-8.16)	1.0000	0.51 (0.05-3.32)	0.1940	3.09 (0.60-16.26)	1.0000	1.50 (0.27-9.18)	1.0000	0.00 (0.00-5.96)	0.5097	0.00 (0.00-3.40)												
Heart failure	0.0175	3.25 (1.31-8.21)	1.0000	0.86 (0.25-3.21)	0.0542	3.85 (1.18-13.07)	0.7164	0.70 (0.19-3.20)	0.6353	2.10 (0.40-12.10)	0.6757	0.56 (0.10-3.22)												
Chronic obstructive pulmonary disease	0.0691	4.48 (1.07-22.19)	0.6142	1.83 (0.36-7.91)	0.4718	1.83 (0.15-12.61)	0.6050	0.45 (0.03-4.19)	0.2870	3.40 (0.42-24.65)	0.5097	0.00 (0.00-3.40)												
History of any smoking	0.5079	1.25 (0.63-2.45)	0.6549	0.77 (0.32-1.89)	0.3615	1.79 (0.52-6.09)	1.0000	0.80 (0.20-3.06)	0.6652	1.83 (0.37-8.72)	0.7172	0.69 (0.18-2.81)												
Hypertension medications																								
ACE-I^e^	0.0798	0.47 (0.22-1.02)	0.5743	0.67 (0.24-2.18)	1.0000	0.86 (0.26-3.15)	1.0000	1.13 (0.21-6.18)	0.2778	0.00 (0.00-2.34)	1.0000	0.71 (0.11-4.55)	0.1017	0.21 (0.03-1.36)	0.1416	0.37 (0.10-1.40)	0.4473	1.86 (0.37-9.27)						
ARB^e^	0.5688	0.77 (0.37-1.60)	0.3236	1.72 (0.63-4.35)	0.4622	0.40 (0.08-1.71)	0.7104	1.58 (0.38-7.77)	1.0000	1.35 (0.25-8.41)	0.6785	1.80 (0.35-7.80)							0.0381	0.09 (0.01-0.88)	0.6901	1.24 (0.43-3.54)	0.3370	0.40 (0.06-2.59)
Selected labs at presentation																								
Potassium [mmol/L]	0.7739	-0.20 (-0.30-0.20)	0.9438	0.00 (-0.03-0.20)	0.6645	0.20 (-0.30-0.60)	0.9366	0.10 (-0.40-0.50)	0.8149	0.10 (-0.40-0.60)	0.8900	0.10 (-0.30-0.40)												
Sodium [mmol/L]	0.0066	-3.00 (-6.00--1.00)	0.1488	-1.00 (-4.00-1.00)	0.6499	1.00 (-2.00-4.00)	0.9597	-1.00 (-4.00-5.00)	0.7677	-3.00 (-7.00-7.00)	0.7423	-1.00 (-3.00-6.00)	0.0081	0.69 (0.52-0.91)					0.0117	0.71 (0.55-0.93)				
Creatinine [mg/dL]	0.1303	0.00 (0-0.40)	0.8258	0.00 (-0.20-0.20)	0.5349	0.00 (-0.20-0.50)	0.6255	-0.10 (-0.30-0.20)	0.2489	0.55 (-0.50-1.90)	0.8395	0.00 (-0.50-0.20)												

a p-values from Fisher’s exact test for categorical variables or Mann-Whitney U test for continuous variables.

b p-values from logistic regression.

c OR indicates ratio of outcome in presence compared to absence of categorical baseline characteristic; difference of medians indicates median of outcome-positive group minus median of outcome-negative group for continous variables.

d compared to room air.

e patients on ARB were excluded from the group compared to patients on ACE-I, and patients on ACE-I were excluded from the group compared to patients on ARB; OR, odds ratio; CI, confidence interval.

Among patients with hypertension, age was independently significantly associated with admission to the hospital, but not admission to the ICU or death (Table 3). Diabetes was independently associated with admission to the hospital and admission to the ICU. CAD was independently associated with death. Low serum sodium concentration at presentation was independently associated with admission to the hospital. ACE-I use was not independently associated with any outcome when analyzed by multivariable logistic regression. ARB use was independently associated with less frequent admission to the hospital (OR 0.09; 95% CI 0.01 to 0.88, *P* = 0.0381), but not admission to the ICU or death. Calculation of E-values to indicate potential unmeasured confounding supports the strength of these results (Supplementary Table 2). These data suggest that ARB medications are associated with a reduced risk of hospitalization and no increased risk of admission to the ICU or death among patients with hypertension and COVID-19.

## Discussion

This retrospective cohort study included a diverse cohort of 1023 outpatient and inpatient individuals with COVID-19 confirmed by RT-PCR. In 550 patients with chart documentation of past medical history, we identified baseline characteristics and comorbidities that represent independent risk factors for admission to the hospital, admission to the ICU, and death. Hypertension was the most common pre-existing diagnosis of COVID-19 patients in this study. Use of ACE-I and ARB medications was each independently associated with a reduced risk of admission to the hospital, but not admission to the ICU or death. When compared to other patients with hypertension not on ACE-I or ARB medications, hypertensive patients on ARB diagnosed with COVID-19 also had a significantly lower risk of hospitalization.

These findings are particularly relevant in the context of recent studies exploring ACE-I and ARB use in COVID-19 and significant controversy as to whether these medications confer benefit or harm in the setting of COVID-19. Previous studies were limited by analyzing only inpatients, failing to analyze the risk of hospitalization among outpatients, analyzing ACE-I and ARB together without distinction, imputing missing data, identifying some COVID-19 patients by chest CT without definitive RT-PCR, and/or excluding some patients based on age. In contrast, the present study analyzed the risk of hospitalization among outpatients, clinical outcomes among inpatients, distinguished between ACE-I and ARB medications, identified all COVID-19 patients by definitive RT-PCR, included all patients regardless of age, and did not impute missing data. Particularly valuable are the findings here that ACE-I or ARB use are each independently associated with reduced risk of hospitalization and no increased risk of any other adverse outcome measured, especially in light of reports calling for use of these medications to be withdrawn due to concern over deleterious effects in COVID-19.^21,28–32^ Moreover, it is possible that different baseline characteristics in the ACE-I and ARB groups understated the beneficial effects of these medications. Taken together, these findings suggest that continued use of ACE-I or ARB may be safe in the setting of COVID-19 and that further investigation of safety and therapeutic effects, especially of ARB, is worthwhile.

The fundamental distinction between ACE-I and ARB mechanisms of action and physiological effects may reflect different effects in COVID-19 that will be elucidated in future prospective studies. Investigation in rats showed that while both ACE-I and ARB increase expression levels of ACE2, only ARB increases ACE2 activity.^13^ Enhanced ACE2 activity may be particularly important in the context of COVID-19 lung injury due to the anti-inflammatory effects of ACE2. In light of these differences, it was essential that we distinguished between ACE-I and ARB use in this study.

Interestingly, ARB or ACE-I use was associated with reduced risk of hospitalization, specifically without reduced risk of admission to the ICU or death. This suggests that in contrast to concerns over upregulation of ACE2 there could be prophylactic effects and/or therapeutic activity in cases of mild disease, in addition to the potential therapeutic effects in moderate to severe disease that have been hypothesized in the literarture. If ACE-I or ARB medicaitons indeed demonstrate prospective benefit in COVID-19 management, the effects may be seen particularly in the outpatient setting, where treatment could reduce disease incidence and/or convert severe courses to a milder form before reaching the levels of disease progression seen in many hospitalized patients; this is consistent with recent observations suggesting that ACE-I or ARB medication use is associated with a decreased incidence of influenza in the United Kingdom.^33^ Only 1 of 10 clinical trials currently evaluating the use of ARB medications in COVID-19 patients include outpatients, while the remaining 9 focus on critically ill hospitalized patients. Thus, there is a largely untapped opportunity to explore the prophylactic and therapeutic effects of ACE-I and ARB medications in the outpatient setting.

This study had several limitations. Although this retrospective cohort analysis used robust statistical methods to account for confounding variables, sample size was limited, treatment was not randomly or blindly assigned, and there are potential unmeasured variables that could have confounded the results. Importantly, as an observational study, the data can only demonstrate association between observed exposures and outcomes without proving causality. While we chose not to collect or adjust for use of non-hypertension medications because none are known to alter disease course or outcomes of COVID-19, it is possible that use of other medications could have affected the results. As for most studies of this nature, hospital admission, ICU admission, and oxygen supplementation were determined at the discretion of the treating physicians rather than a uniform protocol. In addition, there may be prevalent user bias for patients on ACE-I or ARB medications. Any undiagnosed pre-existing conditions or medications not recorded in the medical chart were not identified (ascertainment bias). Chart documentation of past medical history beyond age and sex was not available for patients who received their diagnosis of COVID-19 via drive through testing and had no further care or encounters at Stanford Hospital and Clinics or institutions in the network. We may not have known if any patients died at home, as this may not consistently be documented in the medical chart. In addition, 14 patients remained in the hospital at the time this study was completed. Lastly, the study reflects a patient population predominantly in Northern California; thus, examination of additional populations will be valuable. Despite these limitations, our observational study provides evidence that risk of ICU admission and death is not higher among COVID-19 patients on ACE-I or ARB medications and that risk of hospitalization may be lower among ARB and ACE-I users.

In light of controversy regarding the roles of ACE-I and ARB in COVID-19 and the sparsity of outpatient data to date, this study provides timely evidence to support the continued use of ACE-I or ARB in the setting of COVID-19, suggests the utility of future prospective studies to evaluate the potential efficacy of these medications in COVID-19 disease management, and provides US data on outpatient risk of hospitalization by baseline characteristics and pre-existing conditions.

## Supplementary material

For supplementary material accompanying this paper visit http://dx.doi.org/10.1017/cts.2020.489. click here to view supplementary material
