# Supplementary material for: Safety of ACE-I and ARB medications in COVID-19: A retrospective cohort study of inpatients and outpatients in California
Source: J Clin Transl Sci. 2021 Jun 1;5(1):e8. doi: 10.1017/cts.2020.489 (PMC7605244; doi:10.1017/cts.2020.489)
Supplement: Supplementary file 1 [file S2059866120004896sup001.docx]

**Supplemental Table 1.** E-values for odds ratios from multivariable analyses in Table 2.

|  | ACE-I + confounders | | | | | | | | ARB + confounders | | | | | | | |
| --- | --- | --- | --- | --- | --- | --- | --- | --- | --- | --- | --- | --- | --- | --- | --- | --- |
|  | **Admission to hospital** | | | **Admission to ICU** | | | **Death** | | **Admission to hospital** | | | **Admission to ICU** | | | **Death** | |
| **Baseline characteristic** | Estimate | CI | Estimate | | CI | Estimate | | CI | Estimate | CI | Estimate | | CI | Estimate | | CI |
| Age | 1.24 | 1.16 | 1.11 | | 1.16 | 1.31 | | 1.16 | 1.24 | 1.21 | 1.11 | | 1.11 | 1.34 | | 1.16 |
| Female sex |  |  |  | |  |  | |  |  |  |  | |  |  | |  |
| Body mass index | 1.21 | 1.11 | 1.37 | | 1.16 |  | |  | 1.31 | 1.16 | 1.37 | | 1.16 |  | |  |
| Pre-existing diagnoses |  |  |  | |  |  | |  |  |  |  | |  |  | |  |
| Hypertension |  |  |  | |  |  | |  |  |  |  | |  |  | |  |
| Diabetes | 7.44 | 3.39 | 14.14 | | 5.51 |  | |  | 7.14 | 3.21 | 9.23 | | 3.64 |  | |  |
| Asthma |  |  |  | |  |  | |  |  |  |  | |  |  | |  |
| Cancer |  |  | 5.23 | | 1.37 |  | |  |  |  | 4.64 | | 1.24 |  | |  |
| Coronary artery disease | 3.10 | 2.55 | 1.24 | | 6.87 | 15.02 | | 3.58 | 5.71 | 1.69 | 3.19 | | 2.50 | 16.48 | | 3.43 |
| Autoimmune or autoinflammatory | 3.93 | 1.60 |  | |  |  | |  | 5.27 | 1.49 |  | |  |  | |  |
| Heart failure |  |  |  | |  |  | |  |  |  |  | |  |  | |  |
| Chronic obstructive pulmonary disease |  |  |  | |  |  | |  |  |  |  | |  |  | |  |
| History of any smoking |  |  |  | |  |  | |  |  |  |  | |  |  | |  |
| Medications |  |  |  | |  |  | |  |  |  |  | |  |  | |  |
| ACE-I | 4.08 | 1.21 | 4.70 | | 1.81 | 2.58 | | 4.08 |  |  |  | |  |  | |  |
| ARB |  |  |  | |  |  | |  | 4.57 | 1.46 | 1.34 | | 4.31 | 5.33 | | 3.04 |
| CI, least extreme 95% confidence interval | |  |  | |  |  | |  |  |  |  | |  |  | |  |

**Supplemental Table 2.** E-values for odds ratios from multivariable analyses in Table 3.

|  | ACE-I + confounders | | | | | | ARB + confounders | | | | | |
| --- | --- | --- | --- | --- | --- | --- | --- | --- | --- | --- | --- | --- |
|  | **Admission to hospital** | | **Admission to ICU** | | **Death** | | **Admission to hospital** | | **Admission to ICU** | | **Death** | |
| **Baseline characteristic** | Estimate | CI | Estimate | CI | Estimate | CI | Estimate | CI | Estimate | CI | Estimate | CI |
| Age | 1.40 | 1.16 | 1.21 | 1.11 | 1.24 | 1.32 | 1.46 | 1.24 | 1.11 | 1.21 | 1.28 | 1.21 |
| Female sex |  |  |  |  |  |  |  |  |  |  |  |  |
| Body mass index |  |  |  |  |  |  |  |  |  |  |  |  |
| Pre-existing diagnoses |  |  |  |  |  |  |  |  |  |  |  |  |
| Diabetes | 17.31 | 1.74 | 14.52 | 3.19 |  |  | 8.43 | 2.84 | 6.38 | 1.49 |  |  |
| Asthma |  |  |  |  |  |  |  |  |  |  |  |  |
| Cancer |  |  |  |  |  |  |  |  |  |  |  |  |
| Coronary artery disease |  |  |  |  | 22.41 | 3.41 |  |  | 3.50 | 2.35 | 34.49 | 2.85 |
| Autoimmune or autoinflammatory |  |  |  |  |  |  |  |  |  |  |  |  |
| Heart failure |  |  |  |  |  |  |  |  |  |  |  |  |
| Chronic obstructive pulmonary disease |  |  |  |  |  |  |  |  |  |  |  |  |
| History of any smoking |  |  |  |  |  |  |  |  |  |  |  |  |
| Hypertension medications |  |  |  |  |  |  |  |  |  |  |  |  |
| ACE-I | 8.99 | 2.06 | 4.85 | 2.15 | 3.12 | 4.85 |  |  |  |  |  |  |
| ARB |  |  |  |  |  |  | 21.71 | 1.53 | 1.79 | 4.08 | 4.44 | 32.83 |
| Selected labs at presentation |  |  |  |  |  |  |  |  |  |  |  |  |
| Potassium [mmol/L] |  |  |  |  |  |  |  |  |  |  |  |  |
| Sodium [mmol/L] | 2.26 | 1.43 |  |  |  |  | 2.17 | 1.36 |  |  |  |  |
| Creatinine [mg/dL] |  |  |  |  |  |  |  |  |  |  |  |  |
| CI, least extreme 95% confidence interval |  |  |  |  |  |  |  |  |  |  |  |  |
